# Approaches to Reach Trustworthy Patient Education: A Narrative Review

**DOI:** 10.3390/healthcare12232322

**Published:** 2024-11-21

**Authors:** Xiafei Lyu, Jing Li, Sheyu Li

**Affiliations:** 1 Department of Radiology, West China Hospital, Sichuan University, Chengdu 610041, China; xiafeilyu@wchscu.edu.cn; 2 Department of Endocrinology and Metabolism, MAGIC China Centre, Chinese Evidence-Based Medicine Centre, West China Hospital, Sichuan University, Chengdu 610041, China

**Keywords:** patient education, health behavior, public health, health promotion, ecological model, conflicts of interest

## Abstract

Background: Patient education is a cornerstone of modern healthcare. Health literacy improves health-related quality of life and health outcomes of patients, enhanced by effective patient education. Inadequate competency of patient education in healthcare providers triggered this review to summarize common approaches and recent advancements. Methods: This narrative review summarizes common approaches and recent advancements in patient education with their relations to health literacy, their strengths, limitations, and practical issues. Results: This review highlighted the multifaceted approaches to patient education, emphasizing the importance of tailoring methods to meet the diverse needs of patients. By integrating various strategies, including intrapersonal, interpersonal, and societal/community-level interventions, healthcare providers can create a more comprehensive educational experience that addresses the complexities of patient needs, meanwhile improving the health literacy of patients. With the rise of digital media and artificial intelligence, there is an increasing need for innovative educational resources that can effectively reach and engage patients. Ongoing research and collaboration among healthcare professionals and policymakers will be essential to refine educational strategies and adapt to emerging challenges. It is essential to remain vigilant about potential conflicts of interest that may compromise the integrity of educational content. Conclusion: Effective patient education empowers individuals and their contributions to a healthier society by fostering informed decision-making and encouraging proactive health management.

## 1. Introduction

Patient education is a cornerstone of modern healthcare, enhancing patients’ understanding, promoting engagement, encouraging behavior change, and ultimately improving health outcomes [1]. Effective patient education encompasses a wide range of knowledge, extending beyond an understanding of disease and treatment to include education theory, psychology, communication skills, technology, public health policy, and legal considerations. However, inadequate and inappropriate patient education can lead to misunderstanding, confusion, and adverse health consequences [2]. Health literacy is defined as the individual’s capacity to obtain, process, and understand basic health information and services needed to make appropriate health decisions [3]. Several studies have shown that health literacy is closely related to health-related quality of life and health outcomes of patients [4,5,6]. The prototypical theoretical framework regarding health literacy is that of Nutbeam [7]; it divided health literacy into three dimensions: functional health literacy, which refers to the basic reading and writing skills necessary to understand health information; interactive health literacy, which refers to more advanced cognitive and literacy skills together with social skills to actively participate in health-related discussions; and critical health literacy, which refers to more advanced cognitive skills together with social skills to critically analyze health information, evaluate sources, and make informed decisions about health [7].

Healthcare providers play a pivotal role in conveying information and knowledge to patients during clinical practice. However, many healthcare providers lack adequate competency in patient education, particularly in the areas of communication skills and public health [8,9,10]. Moreover, patient education remains a relatively underdeveloped area in medical training [9], with most healthcare providers expressing a lack of confidence in their ability to tailor educational content to individual patient needs.

Several challenges hinder effective patient education. Health behavior is influenced by numerous individual factors, such as health status, literacy level, learning preferences, personalized needs, disease stage, cultural background, language proficiency, socioeconomic status, and family support systems. These factors complicate the straightforward implementation of patient education interventions. Certain populations, often referred to as “hard to reach”, may face additional barriers due to psychological, demographic, or cultural-environmental factors [11], which can prevent their participation in or benefit from educational programs [12]. Additionally, in the information age, patients are overwhelmed by excessive information, making it imperative for healthcare providers to ensure that patients receive accurate and useful guidance [13]. Some patients may also lack proactivity regarding their health, relying heavily on physicians and being hesitant to independently seek medical information, which further complicates the educational process [14].

Over the years, various methods and approaches to patient education have been developed and refined to meet the diverse needs and preferences of patients [15,16,17,18,19,20,21,22,23,24]. Furthermore, with advancements in technology, the medium for delivering education has expanded from traditional face-to-face interactions to digital health platforms and social media. Consequently, healthcare providers need to employ various strategies and media to improve the health literacy of the patients, ensuring that patients receive the necessary information and skills for making informed health decisions.

Existing educational strategies vary in strengths, limitations, and practical applicability, yet limited guidance is available to help healthcare practitioners tailor these strategies to meet the specific needs of patients with varying health literacy levels. This narrative review aims to address this gap by summarizing commonly used approaches and recent advancements in patient education, analyzing their alignment with health literacy dimensions, and assessing their strengths, limitations, and practical issues. Furthermore, this review seeks to inform policy decision-makers by highlighting the implications of patient education practices. Policymakers can leverage these insights to develop frameworks that promote best practices in patient education across diverse healthcare settings, ensuring accessible, high-quality education for patients with different health literacy levels.

## 2. Methods

We chose the narrative review method in the manuscript because it is commonly employed to synthesize qualitative evidence, offering a more flexible and comprehensive approach to summarizing diverse studies compared to the rigid structure of systematic reviews. This method allows for a broader examination of patient education strategies, considering both the variety of interventions and their evolving nature [25]. We searched PubMed for studies about patient education approaches from September 2013 to September 2024. The search terms for titles, abstracts, or MeSH terms included “patient education” and “health literacy” or their synonyms. Searches were not restricted by language or publication type. The authors added gray literature with their expertise. We also browsed the reference lists of narrative reviews of the interests that were identified in the search.

## 3. Approaches that Target Health Behavior Through Multi-Level Interventions

### 3.1. Ecological Model Addressing the Determinants of Health Behavior

The ecological model is a well-recognized framework for understanding the determinants of health behavior [26]. It proposes that behaviors are complex processes influenced by multiple factors across different levels, and rarely can they be attributed to a single factor. According to this model, multi-level interventions that address factors at various levels are generally more effective in promoting behavior change than those targeting a single level [26].

**Figure 1:**
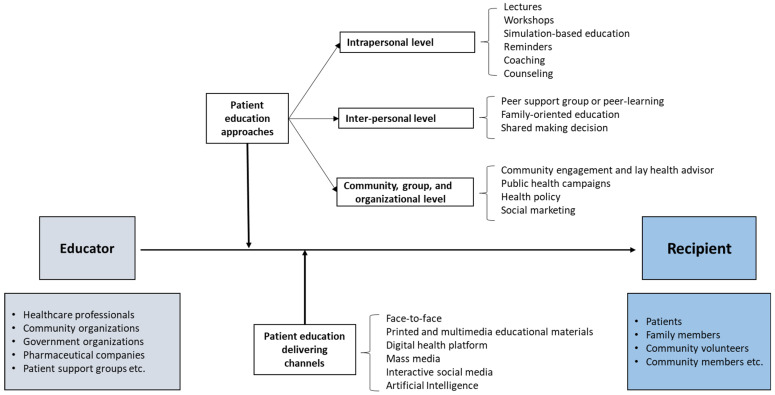
A brief presentation of the methods and media used in patient education.

Aligned with the ecological model, we categorized the patient education interventions into three levels: intrapersonal, interpersonal, and societal. This highlights how interventions can be tailored to address specific determinants of health behavior at each level. Importantly, in real-world practice, these levels often overlap, reflecting the interconnected nature of the factors influencing health behaviors. Patient education approaches are summarized in Figure 1 and Table 1.

### 3.2. Patient Education Approaches Targeted at the Intrapersonal Level

Intrapersonal-level patient education enhances health knowledge, self-efficacy, and behavior change, empowering patients to take a more active role in managing their own health. It is an essential means of improving health outcomes and quality of life. At the intrapersonal level, educational theories commonly employed include the Health Belief Model (HBM), the Theory of Planned Behavior (TPB), and the Transtheoretical Model (TTM) [27]. These theories share key concepts, including beliefs, barriers, and self-efficacy in patient education. Incorporating educational theories into the development of patient education strategies can enhance the formulation process and improve the effectiveness of education [27]. Patient education approaches targeted at the intrapersonal level are as follows.

#### 3.2.1. Lectures and Workshops

Lectures can serve as a patient education approach targeted at either intrapersonal or group levels, depending on the source and size of the audience. Lecturing is a widely used strategy in patient education, especially in scenarios where educators aim to efficiently cover a substantial amount of content [28]. Lectures primarily focus on enhancing functional health literacy by providing essential knowledge, but they can also support interactive and critical health literacy through structured interactions and thoughtful content delivery. In traditional lectures, educators present their educational material while patients listen, followed by a potential exchange of questions and answers, which often focus on clarification rather than in-depth discussion [29]. Studies have shown that lectures can lead to improvements in disease-specific knowledge and quality of life [30]. This traditional method offers several advantages, including efficient information transmission. Lectures can rapidly convey a vast amount of content, making them particularly suitable for situations requiring a quick understanding of basic knowledge, with a volume of information unmatched by other educational methods [31,32]. Therefore, lectures are particularly suitable for chronic diseases with high prevalence, such as diabetes, hypertension, chronic obstructive pulmonary disease, and cancer. Additionally, lectures are typically provided by professional educators, ensuring both accuracy and consistency of content. However, there are notable disadvantages, such as a lack of interaction between patients and educators, an absence of personalized education, and a limited capacity for information absorption [32]. Moreover, the lack of hands-on practice makes it challenging for patients to translate theoretical knowledge into practical skills, highlighting the need to integrate other educational methods to provide a more comprehensive and effective educational experience. To address these limitations, healthcare providers can incorporate greater interactivity, personalized support, and hands-on practice into lectures, overcoming traditional barriers to engagement and practical application. This approach empowers patients to more effectively translate knowledge into actionable health management skills. In practice, healthcare providers can leverage lectures to educate diabetic patients on effective daily dietary management, including guidance on making healthier food choices, balancing carbohydrates, and identifying foods to avoid and prevent blood sugar spikes. Similarly, lectures can be tailored for pregnant women to cover safe and effective exercise across each trimester, offering practical advice on suitable types and intensities of activities to support their health and well-being throughout pregnancy.

In contrast to traditional lecture, which is a passive learning process, an interactive learning workshop is proposed [33]. Workshops primarily enhance interactive and critical health literacy through participatory learning while also addressing functional health literacy by providing practical applications of health information. In the workshops, patients are encouraged to ask questions and are, in turn, asked questions by the educator to foster a deeper understanding of the information presented [34]. Furthermore, patients are encouraged to relate the information to their personal situations, thereby enhancing the application of learned competencies in their daily lives. A considerable amount of time is dedicated to allowing patients to share their personal experiences and discuss them with fellow patients, which helps increase motivation and competence in coping and self-management [30]. However, workshops have some drawbacks. They are typically conducted in small groups, limiting their accessibility to a large patient population. Additionally, organizing workshops requires substantial time, effort, and resources—such as qualified staff, materials, and adequate space—which can be challenges for healthcare providers with limited resources. To address these limitations, healthcare providers can adopt strategies to make workshops more accessible and efficient, such as offering online workshops or training peer educators and volunteers to facilitate certain activities. In practice, healthcare providers can conduct a hypertension prevention workshop, offering heart health knowledge for patients with hypertension or heart disease. The workshop can cover topics such as choosing a low-salt diet, stress management, and daily exercise tips. Patients can participate in group discussions, sharing and learning from each other’s self-management experiences and challenges, as well as discussing how to apply workshop lessons to daily health management.

However, lectures and workshops are not appropriate for acute conditions like acute diseases (e.g., myocardial infarction, acute stroke, acute trauma) and severe infections (e.g., sepsis), as these situations require immediate medical intervention rather than educational knowledge dissemination.

#### 3.2.2. Simulation-Based Education

The simulation-based education has gained widespread recognition within healthcare training and is increasingly embraced in patient education [35]. By utilizing environments designed to mimic real clinical encounters and lifelike experiences, simulation-based education brings theoretical knowledge to life. Simulation-based education can simultaneously enhance functional, interactive, and critical health literacy by providing immersive, hands-on learning experiences that promote practical application and decision-making skills. Compared with traditional approaches, simulation-based patient education has been demonstrated to promote skill mastery, increase patient confidence and self-efficacy, enhance adaptability to complex and emergency situations, and reduce readmissions and healthcare costs [36]. For example, in a randomized clinical trial, simulation-based mastery learning produced superior outcomes in ventricular assist device self-care skills compared to usual training [37]. A higher proportion of participants in the simulation group met the minimum passing standard, demonstrated better power source management, and exhibited improved dressing change skills compared to those in the usual training group [37]. The advantages of simulation-based learning in patient education include the provision of a realistic learning environment, hands-on experience, immediate feedback, and tailored training, all without imposing ethical, economic, or technical risks [19,38,39]. This approach is particularly effective for teaching complex medical procedures, techniques, and emergency situations, such as simulated scenarios for blood glucose testing, insulin administration, severe allergy self-management, and practicing home dialysis techniques [40]. However, simulation-based education also has drawbacks, such as being resource intensive, having limited accessibility, lacking standardization, presenting unrealistic scenarios, and being of a time-consuming nature, which may constrain its effectiveness and application in patient education. To overcome these limitations, healthcare providers can streamline simulation sessions by focusing on shorter, high-impact simulations that target key skills, enabling efficient learning without requiring excessive time commitments. In practice, healthcare providers can use simulation-based education to teach soon-to-be first-time parents how to change diapers, bathe their newborns, and perform other essential caregiving tasks.

#### 3.2.3. Reminders

Reminder-based educational methods are an effective approach for patient education, as they reinforce information, prompt health-related actions, and can be used to promote medication adherence [41], ensure timely vaccinations and screenings [42], manage chronic diseases [43,44], reduce missed appointments [45], and encourage regular physical activity [46]. Reminders primarily enhance functional and interactive health literacy by reinforcing tasks and encouraging engagement. These reminders can be delivered through various means, including technology-assisted systems, mail or email, or phone-based calls or messages. Research has shown that reminders can significantly improve patient adherence to prescribed treatment plans or recommended health practices. For instance, results from a randomized clinical trial demonstrated that reminder calls, combined with low literacy level instruction mail, were associated with a lower proportion of mishandled fecal immunochemical test samples [47]. Additionally, a systematic review found that the use of reminder systems for patients with gestational diabetes mellitus increased patient compliance with blood glucose level monitoring, decreased mean blood glucose level values, and was associated with a decreased risk of cesarean delivery [43]. However, one concern with implementing reminders is that their effectiveness may be compromised if the content is too general. Tailored reminders can address this issue by offering more personalized communication [44]. Reminders are well-suited for chronic conditions (such as diabetes, hypertension, and asthma), vaccination schedules, and other scenarios requiring regular treatment. However, they are less effective for acute illnesses or rapidly progressing conditions, where reminders may not provide timely support. Practical applications of reminders can include notifying patients to undergo regular health check-ups or screenings, such as for breast cancer, cervical cancer, or colorectal cancer, to help detect potential health issues early.

#### 3.2.4. Coaching

Coaching in patient education is a collaborative, patient-centered approach where health professionals act as facilitators, helping patients take control of their health by setting personal goals and developing action plans for effective self-management [48]. Coaching primarily enhances interactive, critical health literacy by encouraging critical thinking and improving comprehension. This intervention not only helps patients acquire knowledge but also guides them in translating this knowledge into actionable behaviors [49,50,51]. In the process of coaching, patients also receive resources that help them overcome challenges and achieve their goals. This support fosters a positive mindset and self-awareness, unlocking patients’ potential to better navigate life’s difficulties and challenges [52]. Coaching has been applied to a wide range of health behaviors, including substance abuse, overeating, and physical inactivity [53]. For instance, a randomized clinical trial demonstrated that a 3-month physical activity coaching intervention led to improved and sustained physical activity and health-related outcomes in adult ambulatory hospital patients at the 9-month follow-up [49]. However, coaching demands high levels of insight and communication skills, as well as significant time and human resources [54]. To address these challenges, healthcare providers can incorporate group coaching sessions and utilize virtual or telehealth coaching. What is more, coaching is not suitable for severe mental illnesses (such as severe depression and bipolar disorder) and terminal cancer, as patients with these diseases often require specialized psychological or palliative treatment, making coaching less effective. In practice, healthcare providers can use coaching to support patients in weight loss by helping overweight or obese patients set realistic weight-loss goals, providing guidance on diet, exercise, and behavioral changes, and assisting them in establishing healthy lifestyle habits.

#### 3.2.5. Personal Counseling

In contrast to coaching, which is primarily action-oriented and emphasizes specific goals, counseling delves deeper into the underlying psychological issues related to a patient’s health behaviors. Counseling is a process that aids in the improvement of an individual’s attitude, behavior, and personality, and it is an important patient education approach in chronic illnesses, where psychosocial factors significantly influence both etiology and disease progression [55]. Personal counseling also primarily enhances interactive, critical health literacy by promoting active engagement, critical thinking, and understanding of health information. Treatment approaches in clinical psychology, such as Cognitive Behavioral Therapy (CBT) and Dialectical Behavior Therapy (DBT), can enhance patient education by providing individuals with essential knowledge and practical skills to manage their mental health [56,57,58]. By incorporating these approaches into patient education, individuals gain self-awareness, improved coping skills, and active engagement in their mental health journey, enabling them to tackle challenges beyond therapy sessions. Commonly used counseling strategies include the Transtheoretical Model (Stages of Change), the Five A’s (Ask, Advise, Assess, Assist, Arrange), FRAMES (Feedback about Personal Risk, Responsibility of the Patient, Advice to Change, Menu of Options, Empathy, Self-Efficacy Enhancement), Motivational Interviewing, and BATHE (Background, Affect, Troubles, Handling, Empathy) [59]. All the counseling strategies provide a systematic strategy for activating patients’ intrinsic motivation and engaging patients in behavior change. According to a randomized clinical trial, counseling using a cognitive behavioral approach was effective in improving self-esteem and body image, which might lead to an increase in exclusive breastfeeding among women [60]. Another randomized clinical trial demonstrated that solution-focused education and counseling intervention decreased problematic Internet usage and increased sleep quality, and the median Internet Addiction Test and Pittsburg Sleep Quality Index scores of the adolescents in the intervention group were significantly lower than those of the controls (*p*  <  0.05) [61]. However, counseling also presents some challenges. It can be resource-intensive, requiring significant time and skilled personnel [62], which may not be feasible in busy healthcare settings. To address these limitations, several strategies can be implemented. Creating structured counseling protocols can help streamline the process, ensuring that essential information is consistently communicated while still allowing for personalization to meet individual patient needs [59]. Implementing group sessions can also provide a cost-effective alternative while still allowing for interactive learning and support among peers [59].

Counseling is suitable for chronic diseases, mental health issues, and lifestyle-related diseases. However, it is not suitable for acute and emergency situations, as these conditions are better managed with rapid professional treatment rather than counseling, which focuses on psychological support and behavioral change. In practice, when patients are diagnosed with cancer, diabetes, or other chronic diseases, health providers can use counseling to help them come to terms with the diagnosis and manage feelings of anxiety, fear, or sadness, enabling better self-management of their condition.

### 3.3. Patient Education Approaches Targeted at the Interpersonal Level

The social support system is not merely an auxiliary role in patient education; it is an essential force in helping patients establish and maintain healthy behaviors. It contributes to enhancing the effectiveness of patient education and ultimately improves patients’ health outcomes. At the interpersonal level, educational theories commonly employed include social cognitive theory, social networks, and social support [63]. These theories share key concepts, including self-efficacy, observational learning or modeling, and social support [63]. Patient education approaches that target the interpersonal level and incorporate these theoretical frameworks are as follows.

#### 3.3.1. Peer Support Group or Peer-Learning

Recent literature has increasingly focused on peer education in the self-management of chronic illnesses [64,65]. Peer education involves the sharing of information among individuals with similar disease experiences, aiming to improve knowledge, attitudes, and behaviors related to disease management [66]. Peer support groups or peer-learning enhances interactive, critical, and functional health literacy by fostering social engagement, encouraging critical discussions, and providing practical knowledge. Additionally, peer education provides patients with essential social support [67]. This approach is particularly effective in delivering tailored health information, which facilitates the translation of knowledge into changes in motivation and beliefs [68]. Currently, peer education is widely implemented across diverse settings to address a broad spectrum of chronic psychological and physical disease management and rehabilitation, especially in cardiovascular disease, diabetes, cancer, HIV/AIDS prevention, adolescent care, and mental diseases [68,69,70,71,72,73]. A systematic review demonstrated that, compared with usual care, peer support group education resulted in better glycemic control and blood pressure management among patients with diabetes and hypertension, respectively. Despite the promising potential of peer-led patient education in healthcare delivery, this approach also presents challenges [74,75]. Barriers include a lack of time and initiative among busy medical personnel to train patient educators [74]. Additionally, concerns have been raised regarding the selection of peer coaches, as the quality and reliability of peer support may vary [74]. Peers without medical qualifications may unintentionally share biased or inaccurate information, leading to confusion or misinformation among patients [65]. However, most of these concerns can be addressed through the integration of professional healthcare supervision and ongoing training for peer educators, ensuring that the contents of peer-led activities are reviewed and approved by medical personnel before delivery [65,76]. Peer support groups or peer-learning are suitable for chronic diseases but are not suitable for acute illnesses and severe intellectual disabilities, as these situations require urgent treatment or specialized care, and patients may find it difficult to participate in or benefit from peer support. In practice, healthcare providers can use peer support groups or peer-learning for the long-term management of patients with pituitary tumors. By creating social media group chats, these patients can share management experiences, coping strategies, and success stories with each other, strengthening their confidence and ability in self-management.

#### 3.3.2. Family-Oriented Education

Family-oriented education shifts the focus of care from the patient alone to a more inclusive model that centers both the patient and their active family members [77]. Family-oriented education primarily enhances all the interactive, critical, and functional health literacy. Involvement and support from family members can enhance patient motivation, increase adaptability to their situations, and ultimately improve health outcomes and quality of life [24,78,79,80,81,82]. Additionally, family involvement allows for the delivery of education that is tailored to the specific needs of the patients [82]. A quantitative systematic review demonstrated that family-oriented education could lead to improvements in readmission rates, emergency department visits, and anxiety levels compared with standard care among patients with chronic diseases [82]. This approach is particularly beneficial for the management of chronic diseases, pediatric and adolescent health, and conditions requiring long-term care. However, while family-oriented patient education offers significant advantages, such as personalized care and robust family support, it also faces challenges, including complex family dynamics and constraints related to time and resources [83]. These challenges need to be carefully considered and effectively managed to optimize educational and support outcomes, for example, by focusing on key caregivers. In practice, healthcare providers can use family-oriented education to educate the parents of children with type 1 diabetes, enabling them to better assist in managing the child’s health.

#### 3.3.3. Shared Decision-Making

Shared decision-making (SDM) is attracting increased interest in healthcare and patient education [1]. SDM is defined as a collaborative communication process in which patients and clinicians work together to make medical decisions that best align with an individual patient’s preferences and values [1]. SDM involves active engagement between healthcare providers and patients, which can commendably enhance the interactive and critical health literacy of the patients. The primary goal of SDM is to empower patients with the essential knowledge and resources to make informed decisions regarding their health, thereby reducing regret or conflict in decision-making, with support from their clinicians [84]. SMD is applicable when a patient faces medical decisions with divergent paths that carry significant and lasting consequences, such as major surgeries or medications, or when choices are influenced by personal values and preferences, particularly in chronic disease management [84]. According to a systematic review, SDM was associated with improvements in participants’ knowledge, accuracy of risk perceptions, and alignment between informed values and care choices compared to usual care [14]. Strategies to enhance the implementation of SMD include training healthcare in communication techniques, engaging multidisciplinary medical teams, incorporating trained decision coaches, and using tools such as patient decision aids tailored to appropriate literacy and numeracy levels [1]. Despite its benefits, SDM has not yet been widely adopted by healthcare professionals. Its implementation requires significant time and resources for in-depth discussions, which may not always be feasible in busy clinical settings [85]. Moreover, patients with limited health literacy may find it challenging to actively participate in SDM [1,85]. To overcome these challenges, health providers can leverage decision aids, such as pamphlets, videos, or digital tools, to provide patients with clear, accessible information. In practice, when patients face a choice between surgical and non-surgical treatments (such as joint replacement surgery or conservative treatment), doctors can use shared decision-making to help patients understand the risks, recovery times, and long-term outcomes of each option, enabling them to choose the approach that best meets their needs.

### 3.4. Patient Education Approaches Targeted at Community, Group, and Organizational Level

Improving health requires understanding the multiple determinants across various levels, extending beyond individual and interpersonal factors to include the macro level, which encompasses communities, groups, and organizations. Patients’ health behaviors are influenced not only by personal health knowledge and social support systems but also by factors such as the accessibility of community health facilities and health-related regulations and policies. Therefore, it is essential to also focus on health education at the community and societal levels. Theories that target these broader macro levels include community engagement, diffusion of innovations, and organizational change [86]. It is important to note that strategies at the macro level typically incorporate health promotion strategies at the individual or intrapersonal level. The following are some health promotion approaches designed to address these broader levels.

#### 3.4.1. Community Engagement and Lay Health Advisor

Community engagement is a concept of empowerment, defined as a process that enables individuals or communities to take control of their lives or environments. In the process of community engagement, community members actively participate in identifying and solving community issues, therefore expanding their capacity to effect desired changes. This process involves not only engaging individuals in actions but also empowering them to allocate resources toward community, policy, and systems-level changes that enhance the overall well-being of the entire community.

One key element of this approach is the selection of lay health advisors (LHAs). LHAs, who are ‘insiders’ with healthcare backgrounds and a deep understanding of the community’s internal mechanisms, strengths, weaknesses, and needs [87,88], play a critical role in chronic disease self-management and health promotion at the community level, such as diabetes [89], hypertension [90], cancer screening [91], and maternal and child health [92]. By providing support and encouragement, lay health advisors help individuals articulate their health concerns and questions, enhancing the interactive health literacy of the patients. They can also offer practical information and resources that improve individuals’ understanding of health-related topics. This intervention strategy has been particularly effective in reaching hard-to-reach and minority populations [91,93,94,95,96], addressing healthcare inequalities [88]. For instance, a randomized controlled trial demonstrated that the LHAs strategy had a significantly positive effect on mouth self-examination in remote Aboriginal communities, which were hard-to-reach populations, with participants in the intervention group being 2.04 times more likely to conduct monthly mouth self-examinations compared to those in the control group [96]. A pilot randomized controlled trial investigated the educational impact of LHAs in patients with low health literacy and poorly controlled type 2 diabetes from socioeconomically disadvantaged communities, which were also hard-to-reach populations, compared to usual care; the result demonstrated that the LHAs intervention significantly improved mental health (*p* = 0.049) and illness perception (*p* = 0.040), along with lower resource use, better self-care management, and an improved quality-adjusted life year (QALY) profile at the 7-month follow-up [97].

However, integrating LHAs into the healthcare system presents several challenges, primarily related to the credibility and quality of training [92]. Patients may question the credibility of the advice provided by LHAs, and the training programs available for LHAs may be insufficient, of poor quality, or lack flexibility, particularly in areas requiring counseling, communication skills, and the handling of complex health issues [92]. Additionally, managing emotional relationships and maintaining appropriate boundaries with recipients can also be challenging for LHAs [92]. Therefore, incorporating LHAs into the healthcare system necessitates continuous evaluation and quality improvement to ensure their effectiveness and reliability [88]. Community engagement and lay health advisors are particularly effective for educating patients on community-wide health issues and reaching hard-to-reach populations.

#### 3.4.2. Public Health Campaigns

Public health campaigns are widely recognized as an effective health promotion strategy. By using simple language and engaging visuals, these campaigns enhance the ability of individuals to read, understand, and act on health-related issues, thereby improving their functional health literacy. They are typically initiated by a range of organizations, including government agencies, non-governmental organizations such as the World Health Organization (WHO) and the Red Cross, and health advocacy groups such as the Chinese Diabetes Society, which launched the ‘Blue Light Action’ campaign to raise awareness and promote diabetes prevention [98]. Public health campaigns serve various purposes, including increasing vaccine coverage [99], enhancing cancer screening [100], encouraging healthy behavioral changes [101,102], raising awareness of specific health issues [103], and advocating for health equity [104]. A notable example in China is the Healthy China Initiative (2019–2030), launched by the People’s Republic of China in 2016, which aims to improve the health literacy of the entire population, promote healthy lifestyles, and control risk factors associated with chronic diseases [105]. These campaigns utilize various mediums to disseminate health messages, including television, radio, printed education materials, social media, and community events. The success of public health campaigns often depends on the collaboration among government agencies, medical professionals, mass media, and community organizations.

Public education campaigns have been shown to effectively shape health-related behaviors. For instance, in response to the COVID-19 pandemic, the US Department of Health and Human Services launched the “We Can Do This” public education campaign in April 2021, which increased the likelihood of first-dose COVID-19 vaccination by 125% [106].

Screen use among young children is highly prevalent, disproportionately higher among children from lower-income families and racial/ethnic minorities, and may adversely impact physical and mental health [107]. To help reduce screen time in children, we can launch a health advocacy campaign promoting reduced screen use. This campaign could reach parents and children through various platforms, such as TV, social media, school bulletins, and community boards, to raise awareness of the health impacts of excessive screen time. Local community health centers or schools could distribute free “Screen Time Guide” booklets with recommendations for age-appropriate screen time, alternative activity ideas, and tips for creating a family screen time plan.

The reach and frequency of these campaigns are critical determinants of their impact on the target audience [108], underscoring the need for significant funding to ensure their effectiveness. To address the challenges, healthcare providers can utilize free social media or websites to improve the cost-effectiveness of the activity.

#### 3.4.3. Health Policy

Research in health policy and systems is garnering increased funding and attention as an essential element in global health system strengthening efforts. Unlike public health campaigns, which rely on voluntary public engagement and educational efforts, health policies are typically legally binding and enforceable. By ensuring that information is clear and accessible, health policies can improve individuals’ understanding of health services, rights, and responsibilities, thereby enhancing functional health literacy. These policies are usually initiated by governmental entities such as legislative bodies, executive agencies, or institutions like the Centers for Disease Control and Prevention (CDC). Health policy, as a population-level health promotion approach, is particularly effective in scenarios where individual behaviors have significant public safety implications, for example, disclosure and vaccination during the COVID-19 epidemic, a smoking ban in public places, and mandatory seat belt wearing. Regulation or legislation has proved to be an effective method for health promotion. For example, following the implementation of a nationwide smoking ban in Denmark on June 6, 2007, data indicated a decline in the prevalence of current smokers among both sexes from 2005 to 2010 [109]. Specifically, the current smoking rate among women dropped from 24% to 15%, while among men, it dropped from 27% to 16% [109]. An example of a specific policy recommendation to enhance patient education practices is to incorporate patient education into general practice [110]. Policies could mandate a set number of educational sessions led by healthcare providers for newly diagnosed patients with chronic diseases. By making patient education a standardized part of care, all patients would receive consistent, essential health information, reinforcing self-management behaviors and promoting better health outcomes.

For future policy directions, consider making health education a mandatory course in primary and secondary schools to instill health knowledge early and help young people build a foundation of healthy habits [111]. Develop age-appropriate materials and extracurricular activities focused on areas such as nutrition, healthy life habits, and first aid skills to enhance students’ overall health literacy.

However, using regulation or legislation as a method of patient education also presents some notable drawbacks, particularly concerning ethical issues related to autonomy [112]. Mandating certain behaviors through policy can conflict with individual rights, leading to ethical dilemmas. Additional ethical concerns include balancing risks and benefits and ensuring justice in the application of these policies [112]. Therefore, it is essential to thoroughly understand the determinants of the relevant health behaviors before formulating health policies [113].

#### 3.4.4. Social Marketing

Social marketing is a systematic approach that applies commercial marketing techniques to plan, implement, and evaluate programs aimed at influencing voluntary behavior to enhance individual and social welfare [27]. Social marketing campaigns aim to disseminate clear and straightforward health information to the public, thereby enhancing their functional health literacy. Social marketing is suitable for patient education on health-related behaviors or diseases that have significant public health implications. The core principles of social marketing include focusing on behavior outcomes, prioritizing consumer benefit, and adopting a marketing perspective that differentiates it from other health interventions [27]. This perspective emphasizes tailoring interventions to meet the needs or desires of the target audience and considers the dissemination of information akin to marketing a product. It acknowledges that interventions compete in a dynamic marketplace of ideas and choices [27].

Social marketing operates within three domains: the social–political environment, the health service delivery system, and community and household interactions. It may even involve retail store employees in health education efforts. An example of its application is the improving contraceptive method mix in Indonesia (ICCM) project [114], which aimed to shift focus from shorter-acting to long-acting and permanent contraceptive methods by altering upstream conditions, such as supply chain and policy advocacy, alongside downstream efforts to support informed decision-making among couples. After increasing family planning funding and implementing supportive policies, the project increased 12–31% of long-acting and permanent contraceptives among married women. Although effective, implementing a social marketing strategy can be resource-intensive, requiring significant time and funding, and may face administrative challenges, deterring many health educators from its use. To address the challenges, healthcare providers can also utilize free social media or websites to improve the cost-effectiveness of the activity.

## 4. Channels and Media for Delivering Patient Education

Traditionally, patient education has relied on face-to-face interactions or printed materials. However, advancements in internet technologies have introduced various educational mediums, including digital platforms and interactive social media, offering us a broader array of channels for disseminating patient education resources.

### 4.1. Face-to-Face

Face-to-face education remains one of the most prevalent methods in healthcare systems. The popularity of face-to-face health waned over time but still remains most preferred by both clinicians and patients [115,116]. This approach allows patients to directly address their questions or concerns with health providers, who, in turn, can correct any misinformation and foster a dynamic, ongoing relationship with their patients [79]. Face-to-face patient education is particularly suitable for dealing with complex health issues that require empathetic communication and in-depth discussions [117], as well as emergent situations that necessitate immediate feedback and adaptation [116]. However, this approach demands the physical presence of both patients and healthcare providers, which can strain healthcare resources. It requires substantial staff time and effort, and scheduling can be challenging, particularly in busy or understaffed facilities [118]. Furthermore, not all patients can easily attend in-person educational sessions due to geographical constraints, transportation difficulties, mobility limitations, or conflicting work commitments [119]. These barriers can restrict access to essential education for certain patient populations.

### 4.2. Printed and Multimedia Educational Materials

The development of patient education materials is crucial in enhancing patient education efforts [120]. A randomized, single-blind study demonstrated that providing both written and verbal health information significantly increased caregivers’ burn-care-related knowledge compared to verbal information only [121]. According to the American Medical Association (AMA), educational materials should be written at or below a sixth-grade reading level to ensure accessibility across various literacy levels and to avoid misinformation [122]. However, many online patient education materials are consistently written at a level too high for broad patient comprehension, with great variations in quality. Research has shown an inverse relationship between readability and reliability, where the most readable educational materials often lack reliability, typically coming from commercial and non-profit sources, whereas the most reliable materials are often from lay press sources [122]. Even patients with high health literacy prefer and more easily understand simplified language [123].

Education materials are generally categorized into verbal, written, and multimedia-based formats (including television, computer, and other audiovisual methods) [124,125]. Multimedia-based materials have been found to be more effective than verbal or written formats in improving patient knowledge and behavior [125,126]. Efforts should focus on optimizing the benefits patients derive from multimedia-based education tools. Patient-version clinical practice guidelines (PVGs) present a specific type of patient educational material that translates clinical practice guidelines (CPGs) designed for health professionals into more accessible formats for patients and the public [127]. The involvement of patients in developing these educational materials is crucial for their success [128].

Printed and multimedia educational materials can sometimes fall short in patient education due to a limited understanding of specific patient needs, like those associated with Special Needs Plans (SNPs). Customizing educational content to reflect the unique health conditions and requirements of SNP beneficiaries can reduce misunderstandings and improve the relevance and reliability of the information provided to all patient groups.

### 4.3. Digital Health Platform

The COVID-19 pandemic has reduced face-to-face primary care visits but also accelerated the adoption of digital health initiatives [129]. Common digital health platforms include telephone, short messages, web-based services, mobile medical applications, and virtual health platforms [130]. The widespread adoption of wearable devices, such as smartwatches, fitness trackers, and continuous glucose monitors, offers both patients and healthcare providers real-time insights into key health metrics. When paired with educational content in connected apps, these devices help patients interpret their health data and understand the impact on their overall health, promoting adherence to lifestyle changes. By providing an accessible and interactive communication channel between patients and healthcare providers, digital health platforms hold significant potential for enhancing patient education [131].

A systematic review found that app-based medication adherence interventions positively impacted patient adherence [132]. A study examining the use of the digital prenatal health platform Maven found that 5.32% of users reported it helped them avoid in-person care during pregnancy [133]. Notably, 82.5% of those avoiding in-person visits indicated that Maven enhanced their understanding of warning signs, while 66.1% learned medically accurate information. Adjusted logistic regression analysis revealed a dose-response relationship between the level of digital prenatal health platform Maven use and in-person care avoidance, with higher usage associated with increased odds. Users were more likely to avoid in-person care if they reported that Maven helped them recognize warning signs (adjusted odd ratio 3.55, 95% CI 2.60–4.94) or learn medically accurate information (adjusted odd ratio 2.05, 95% CI 1.59–2.67) [133].

However, the effectiveness of digital health platforms depends on access to the internet or smartphone, which may limit their use among certain populations, particularly those less familiar with digital technology [131]. Furthermore, the vast amount of information available on the digital platform can overwhelm patients, with varying information quality complicating the search for accurate and reliable medical guidance [134]. To address these limitations, healthcare providers can consider designing digital platforms with user-friendly interfaces and clear categories, making them accessible even for those less familiar with technology and highlighting reliable content to guide users toward trustworthy information.

Educators can utilize these platforms to distribute relevant patient education materials, send reminders for medical actions, and facilitate interactive communication. Patients can ask questions, and educators can respond either in real time or during scheduled sessions [131]. Additionally, digital health platforms can be used for online coaching, telerehabilitation, and telemonitoring [135,136].

### 4.4. Mass Media

Mass media effectively reaches large populations or sub-groups, enabling rapid dissemination of health information. Common types of mass media include television, movie, radio, print, digital media (such as online news sites, social media platforms, and video-sharing websites), and outdoor advertising [108]. Mass media plays a crucial role in shaping public opinion and influencing both individual and societal health behaviors. For instance, a systematic review demonstrated that higher exposure to smoking in movies was associated significantly with a 46% increased risk of initiating smoking in adolescents (RR = 1.46; 95% CI = 1.23–1.73) [137]. Being exposed to radio messages about malaria prevention measures among pregnant women in Uganda is linked to a 17.2% increase in awareness and knowledge about the proper use of insecticide-treated bed nets for preventing malaria [138]. Additionally, the “This girl can” physical activity and sports mass media campaign in Australia led to a modest increase of 0.19 days in weekly physical activity [101].

However, there are also some drawbacks to using mass media for health education. The mass media ecosystem is cluttered with competing messages and misinformation, necessitating a discerning audience [137]. Moreover, the high cost of mass media campaigns can strain public health budgets and limit their sustainability [108]. Focusing on the health effects of mass media can optimize resource allocation and advance public health equity.

In practice, healthcare providers can place public health ads on television and radio, and they can also use large outdoor billboards, subway ads, or bus stop posters to display public health information, for example, promoting the importance of preventive screenings (such as breast and colorectal cancer screenings) and promoting the importance of quitting smoking.

### 4.5. Interactive Social Media

Social media platforms such as Facebook, Instagram, Twitter, YouTube, TikTok, and WeChat are increasingly utilized for patient education [139]. They offer a dynamic space for disseminating health information, fostering patient engagement, and facilitating peer-to-peer communication. Public health authorities, health promotion agencies, and non-governmental organizations find social media particularly attractive for reaching diverse target populations due to its broad and deep penetration [140]. As of July 2024, there were 5.17 billion social media users globally, representing 63.7% of the total population, with an average of 8.9 new users per second. On average, users engage 6.7 different social platforms each month, spending about 2 h and 20 min daily on social media [141].

The widespread use and low cost of social media make it one of the most influential platforms for health behavior interventions. It offers the advantage of overcoming physical distance and time barriers, enabling people to seek health information and social support virtually. The effectiveness of social media in patient education has been demonstrated in areas such as diabetes self-management, smoking cessation, cardiovascular diseases, enhancing patient engagement, adherence to treatment, health behavior, and overall well-being [140]. The social media digital platform was effective at enhancing the knowledge, attitudes, and self-care activities of patients, especially helpful for patients with low health literacy [139]. Beyond merely disseminating health information, social media also offers virtual social networks that can support and encourage health behavior change. What is more, social media may be a promising education delivery channel to mitigate health inequities [142]. A systematic review of studies showed that social media interventions for HIV/AIDS prevention had been effective in promoting HIV testing in both high- and middle-income countries [139]. However, challenges persist, including misinformation, data overload, and patient privacy concerns. Social media was a major conduit for spreading misinformation during the COVID-19 pandemic [13,139]. To address the spread of false health information on social media, we recommend strategies such as improving health literacy, using artificial intelligence (AI) tools for misinformation detection, collaborating with social media platforms, and establishing trustworthy sources of information for patients. Moreover, the public or semi-public nature of social media platforms raises significant privacy risks. Patients may inadvertently disclose sensitive personal details, which can be misused, leading to potential privacy breaches both online and offline [143].

In practice, with the patient’s informed consent, healthcare providers can share patients’ recovery stories and experiences on social media to inspire others to actively manage their health. Users can comment, ask questions, or share their own stories, creating a supportive community.

### 4.6. Artificial Intelligence

AI is a rapidly evolving field of computer science that aims to enable a computer algorithm to perform tasks typically associated with human intelligence [144]. Nowadays, AI is widely used in many areas of life, including patient education [145]. AI-based chatbots offer advantages in patient education by providing 24/7 availability, personalized interactions, tailored educational materials, and instant feedback [145]. Studies also showed that patients had a favorable reception for AI with good satisfaction with the response [146,147]. AI can fortify the nexus between patients and healthcare professionals, thereby improving the overall efficacy of patient care [146]. According to a randomized clinical trial, compared with traditional educational materials, an artificial intelligence-enabled patient decision aid showed better decisional quality (K-DQI mean difference, 20.0%; 95% CI, 14.2%-26.1%), collaborative decision-making (CollaboRATE, 8 of 69 [12%] vs. 28 of 60 [47%] patients below median), and satisfaction (numerical rating scale, 9 of 65 [14%] vs. 19 of 58 [33%] patients below median) without significantly affecting consultation time or treatment concordance [148]. However, several limitations of AI in this context must be addressed. A crucial ethical consideration in the application of AI in healthcare is the widespread issue of algorithmic bias and its effects on fairness and equity in healthcare delivery [149]. AI algorithms can be biased in various ways, including racial, gender, and socioeconomic biases. These biases may arise from training datasets that inadequately represent diverse patient populations or from design flaws in the algorithms themselves that lead to discriminatory outcomes [18,149,150]. To reduce bias in AI algorithms, collaborative efforts are needed across several areas, including data collection, algorithm development, and model evaluation [151]. Healthcare organizations should implement mechanisms for continuous monitoring and assessment of AI applications involving multidisciplinary teams of clinicians, data scientists, ethicists, and policymakers. This approach will help evaluate algorithm performance, identify potential bias-related risks, and facilitate necessary corrective actions [149]. Another significant limitation of AI chatbots in responding to health-related questions is their inability to provide detailed references or citations for the information they generate [152]. This lack of transparency can create skepticism and undermine trust in AI-driven healthcare technologies, which may hinder their adoption and acceptance in clinical practice [149]. Concerns also arise in the readability of the answers provided by AI [153]. The complexity of AI-generated answers may be challenging for patients to understand, often written at a college reading level, which is too high for the general population [154]. It is essential to develop user-friendly AI applications that prioritize clarity, using simple language and clear visuals while offering information in various formats (such as audio and video) to accommodate different levels of health literacy. In conclusion, the advantage of AI in this context is its capability to manage routine, time-consuming tasks, enabling clinicians to concentrate on more complex decision-making processes that necessitate human insight and empathy, provided it is utilized as a supportive tool rather than a standalone decision-maker [149].

## 5. Obstacles that Hinder the Effective Delivery of Healthcare Information

There are several obstacles that prevent the effective delivery of healthcare information, including language barriers, variations in healthcare regulations across countries, technical limitations, and privacy concerns, especially with regard to health information exchange. Language barriers can significantly hinder patient comprehension, especially for non-native speakers when dealing with complex medical topics and terminology [155]. Healthcare regulations can differ widely in areas such as patient data privacy, permitted treatments, and standards for health information dissemination, making it challenging for healthcare providers and educators to maintain consistent messaging and share evidence-based practices internationally [156]. Technical limitations can significantly hinder the effective delivery of healthcare information by affecting accessibility, timeliness, and accuracy of information for patients and providers. Certain populations, often referred to as “hard to reach”, such as people with low digital literacy, non-native speakers, or those with low health literacy, may face additional barriers due to psychological, demographic, or cultural–environmental factors [11], which can prevent their participation in or benefit from educational programs [12]. The “hard-to-reach” population may need more comprehensive strategies tailored to their specific conditions; for example, patients with low health literacy may have difficulty understanding medical terminology, so during the educational sessions, health providers should use plain language in all written and verbal communications, avoid complex medical jargon and provide clear, concise explanations. They can also utilize visual aids, infographics, and interactive tools to convey health information effectively. For non-native speakers, healthcare providers can adopt the strategy of using lay health workers. These lay health workers are “insiders” within the non-native-speaking community, possessing healthcare backgrounds and a deep understanding of the community’s internal dynamics, strengths, weaknesses, and needs. They can play a crucial role in patient education for this “hard-to-reach” population [97].

## 6. Example of Patient Education for Active Living that Utilizes Theoretical Frameworks and Planning and Implementation Models

**Figure 2:**
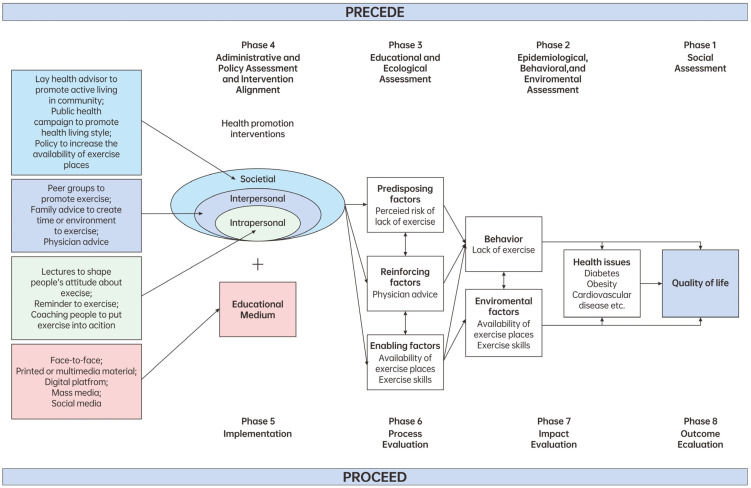
Example of patient education for active living using theoretical frameworks and planning and implementation models.

In the following paragraph, we will illustrate how to apply the ecological model and various patient education techniques discussed above in patient education of a specific disease. We will use the promotion of healthy physical activity as an example to demonstrate this content. The ecological model, a well-established theoretical framework in health promotion, provides a comprehensive understanding of the multiple factors influencing health behavior [26]. It underscores the need to address not only individual knowledge but also social support, community resources, and policies that influence patient behavior. The PRECEDE-PROCEED (PRECEDE stands for Predisposing, Reinforcing and Enabling Constructs in Educational Diagnosis and Evaluation, and PROCEED stands for Policy, Regulatory, and Organizational Constructs in Educational and Environmental Development) model is a widely used framework in public health and health promotion for the systematic planning, implementation, and evaluation of health programs [27]. By integrating these two models, we propose a comprehensive approach to planning and implementing patient education for active living (Figure 2). Both individual habits and environmental factors contribute to inadequate physical activity, with a sedentary lifestyle leading to various chronic diseases, including obesity, diabetes, and cardiovascular conditions, which ultimately diminish quality of life. To address this issue, patient education can be tailored by focusing on targeting predisposing, reinforcing, and enabling factors that affect healthy behaviors. Using the ecological model, education can be delivered at multiple levels, including intrapersonal coaching, peer group support at the interpersonal level, and societal engagement through lay health advisors. Additionally, various educational modalities, such as face-to-face coaching or telehealth coaching, can be employed based on the specific context and needs.

## 7. Conflicts of Interest (COIs) in Patient Education

Conflicts of interest in real-world patient education are unavoidable and can impact the objectivity and accuracy of the information provided to patients [157]. These conflicts may arise from sponsorship by pharmaceutical or medical device companies, healthcare professionals’ financial interests, affiliations with academic or research institutions, or personal biases [157]. Patients usually have a limited ability to identify and assess the influence of potential COIs on the educational content compared to medical professionals [158]. Furthermore, many patient education programs are developed without undergoing an independent peer-review process [158], making them more susceptible to bias. To address this issue, it is essential to disclose all COIs in the development and distribution of patient education programs, including but not limited to funding resources and financial support for practitioners [159]. Government or medical professional societies, due to their non-profit nature, are better suited to oversee or fund such programs to ensure integrity and objectivity [122].

## 8. Conclusions

In conclusion, effective patient education is crucial for enhancing patient engagement, empowerment, and overall health outcomes. This review highlights the multifaceted approaches to patient education, emphasizing the importance of tailoring methods to meet the diverse needs of patients. By integrating various strategies, including intrapersonal, interpersonal, and societal/community-level interventions, healthcare providers can create a more comprehensive educational experience that addresses the complexities of patient needs, meanwhile improving the health literacy of patients. As the healthcare landscape continues to evolve, particularly with the rise of digital media and artificial intelligence, there is an increasing need for innovative educational resources that can effectively reach and engage patients. By keeping abreast of recent advancements in health education, we can optimize patient comprehension and engagement, ultimately leading to better health outcomes. Looking ahead, ongoing research and collaboration among healthcare professionals and policymakers will be essential to refine educational strategies and adapt to emerging challenges. To advance the field of patient education, it is essential to remain vigilant about potential conflicts of interest that may compromise the integrity of educational content. Transparency in the development and dissemination of educational materials will help foster trust between patients and healthcare providers.

In summary, effective patient education not only empowers individuals but also contributes to a healthier society by fostering informed decision-making and encouraging proactive health management. By prioritizing patient education and embracing innovative approaches, we can significantly improve the quality of care and health outcomes for all patients.

## Figures and Tables

**Table 1:** Summary of the main strategies and media for patient education.

Patient Education Approaches Targeted at Intrapersonal Level			
Approaches or Media	Characteristics	Advantages	Limitations
**Lectures**	Educators present their educational material while patients listen, followed by a potential exchange of questions and answers, which often focus on clarification rather than in-depth discussion	Efficient information transmission; provided by professional educators, ensuring both accuracy and consistency of content	Lack of interaction between patients and educators; absence of personalized education; limited capacity for information absorption; lack of hands-on practice
**W** **orkshops**	An interactive learning process where patients are encouraged to ask questions and are, in turn, asked questions by the educator	Foster a deeper understanding of the information presented; relate the information to patients’ personal situations; allowing patients to share their personal experiences and discuss them with fellow patients	Typically conducted in small groups, limiting their accessibility to a large patient population; requires substantial time, effort, and resources
**Simulation-based education**	By utilizing environments designed to mimic real clinical encounters and lifelike experiences, simulation-based education brings theoretical knowledge to life, which is particularly effective for teaching complex medical procedures, techniques, and emergency situations.	Provision of a realistic learning environment; hands-on experience; immediate feedback; tailored training; without imposing ethical, economic, or technical risks	Resource intensive; limited accessibility; lacking standardization; presenting unrealistic scenarios; time-consuming
**Reminder** **s**	Reminders reinforce information, and they can be delivered through various means, including technology-assisted systems, mail or email, or phone-based calls or messages	Prompt health-related actions	The effectiveness may be compromised if the content of the reminder is too general
**C** **oaching**	A collaborative, patient-centered approach where health professionals act as facilitators, helping patients take control of their health by setting personal goals and developing action plans for effective self-management	This intervention not only helps patients acquire knowledge but also guides them in translating this knowledge into actionable behaviors	Coaching demands high levels of insight and communication skills, as well as significant time and human resources
**Counseling**	Counseling delves deeper into the underlying psychological issues related to a patient’s health behaviors	This intervention helps individuals gain self-awareness, improved coping skills, and active engagement in their mental health journey	Resource-intensive, requiring significant time and skilled personnel
**Patient education approaches targeted at the** **Interpersonal level**			
**Peer support group or peer-learning**	Peer education involves the sharing of information among individuals with similar disease experiences, aiming to improve knowledge, attitudes, and behaviors related to disease management	Providing patients with essential social support	A lack of time and initiative among busy medical personnel to train patient educators; the quality and reliability of peer support may vary
**Family-oriented education**	Family-oriented education shifts the focus of care from the patient alone to a more inclusive model that centers both the patient and their active family members	Personalized care and robust family support	Complex family dynamics and constraints related to time and resource
**Shared decision-making**	A collaborative communication process in which patients and clinicians work together to make medical decisions that best align with an individual patient’s preferences and values	Involvement of patients in medical decisions	Its implementation requires significant time and resources for in-depth discussions; requires patients with abundant health literacy
**Patient education approaches targeted at community, group, and organizational level**			
**Community engagement and lay health advisor**	A process that enables individuals or communities to take control of their lives or environments. One key element of this approach is the selection of lay health advisors	Active participation of community members; useful for the education of hard-to-reach people	Credibility and quality of training of the lay health advisors
**Public health campaigns**	Typically initiated by a range of organizations, including government agencies and non-governmental organizations, to improve the awareness of disease	It utilizes various mediums to disseminate health messages that can reach a large group of people	Requires significant funding to ensure their effectiveness
**Health policy**	Health policies are typically legally binding and enforceable for typical health behavior, and they are particularly effective in scenarios where individual behaviors have significant public safety implications	Legally binding and enforceable	Concerning ethical issues related to autonomy
**Social marketing**	Social marketing is a systematic approach that applies commercial marketing techniques to plan, implement, and evaluate programs aimed at influencing voluntary behavior to enhance individual and social welfare	Tailoring interventions to meet the needs or desires of the target audience; acknowledgement that interventions compete in a dynamic marketplace of ideas and choices	Resource-intensive, requiring significant time and funding, and may face administrative challenges
**Channels and media for delivering patient education**			
**Face-to-face**	One of the most prevalent methods in healthcare systems	Most preferred by both clinicians and patients; allowing patients to directly address their questions or concerns with health providers; particularly suitable for dealing with complex health issues and emergent situations	It requires substantial time and effort from both health providers and patients
**Printed and multimedia educational materials**	Education materials are generally categorized into verbal, written, and multimedia-based formats; patient-version clinical practice guideline presents a specific type of patient educational materials	Help patients understand health information with multimedia-based formats	Various reliability and the readability of most educational materials is low
**Digital health platform**	Common digital health platforms include telephone, short messages, web-based services, and mobile medical applications	Providing an accessible and interactive communication channel between patients and healthcare providers	Requirement of access to the internet or smartphone, which may limit their use among certain populations, particularly those less familiar with digital technology; the vast amount of information available on the digital platform can overwhelm patients, with varying information quality complicating the search for accurate and reliable medical guidance
**Mass media**	Common types of mass media include television, movie, radio, print, digital media (such as online news sites, social media platforms, and video-sharing websites), and outdoor advertising	It effectively reaches large populations or sub-groups, enabling rapid dissemination of health information	The mass media ecosystem is cluttered with competing messages and misinformation, necessitating a discerning audience; the high cost of mass media campaigns can strain public health budgets and limit their sustainability
**Interactive social media**	Social media platforms such as Facebook, Instagram, Twitter, YouTube, TikTok, and WeChat	Social media is particularly effective for reaching diverse target populations due to its broad and deep penetration	Misinformation, data overload, and patient privacy concerns
**Artificial intelligence**	AI is a rapidly evolving field of computer science that aims to enable a computer algorithm to perform tasks typically associated with human intelligence	Providing 24/7 availability, personalized interactions, tailored educational materials, and instant feedback	Algorithmic bias; inability to provide detailed references or citations for the information they generate; the complexity of AI-generated answers

## Data Availability

Not applicable.
